# Targeting psoriatic inflammation with natural compounds: mechanistic insights and therapeutic promise

**DOI:** 10.1007/s10787-025-01851-6

**Published:** 2025-07-21

**Authors:** Aya M. Mustafa, Ahmed M. Atwa, Ali M. Elgindy, Mahmoud Abdelrahman Alkabbani, Kawther Magdy Ibrahim, Manar M. Esmail, Riham A. El-Shiekh, Esraa M. Mohamed, Kamel Mahmoud Kamel

**Affiliations:** 1 Department of Pharmacology and Toxicology, Faculty of Pharmacy, Egyptian Russian University, Cairo, Egypt; 2 Department of Pharmacology and Toxicology, College of Pharmacy, Al-Ayen Iraqi University, Thi-Qar, Nasiriyah, 64001 Iraq; 3 Pharmacognosy Department, Faculty of Pharmacy, Cairo University, Cairo, 11562 Egypt; 4 Department of Pharmacognosy, College of Pharmaceutical Sciences and Drug Manufacturing, Misr University for Science and Technology (MUST), Giza, 12585 Egypt

**Keywords:** Psoriasis, Phytotherapy, Anti-inflammatory, Oxidative stress, Immunomodulation

## Abstract

Psoriasis is a chronic immune-mediated skin disorder characterized by aberrant keratinocyte proliferation, immune cell dysregulation, and sustained inflammation driven by cytokines, such as TNF-α, IL-17, and IL-23. Despite advancements in biologic therapies, limitations related to cost, safety, and resistance have prompted interest in alternative strategies. This review explores the pharmacological basis of natural products as promising anti-psoriatic agents, focusing on compounds with multi-targeted mechanisms including anti-inflammatory, anti-oxidant, anti-proliferative, and immunomodulatory activities. Key phytochemicals, such as curcumin, thymoquinone, glycyrrhizin, and boswellic acids, are examined for their roles in modulating psoriatic pathways like NF-κB, IL-23/Th17 axis, and oxidative stress. Evidence from preclinical and clinical studies highlights their potential in reducing psoriasis area and severity index (PASI) scores, mitigating immune hyperactivity, and enhancing the safety and efficacy of standard therapies. Despite promising outcomes, translational hurdles persist, including extract standardization, pharmacokinetic limitations, and regulatory barriers. The integration of omics-based research and advanced formulation technologies is essential to support the clinical application of these agents. This review underscores the therapeutic potential of natural compounds as viable complements or alternatives in modern psoriasis management.

## Introduction

Psoriasis is a chronic, immune-mediated inflammatory skin condition that affects 0.5–3% of the global population (Ibezim et al. 2021). It is marked by epidermal hyperproliferation, aberrant keratinocyte differentiation, and a sustained inflammatory response involving a complex interplay between the innate and adaptive immune systems (Srivastava et al. 2019). The disease manifests in various forms, including plaque, guttate, pustular, inverse, and erythrodermic psoriasis, each with distinct clinical features but overlapping immunopathogenic mechanisms (Reynolds et al. 2022).

Central to the pathogenesis of psoriasis are dysregulated immune responses mediated by dendritic cells, Th1, Th17, and Th22 lymphocytes, and the overproduction of cytokines such as TNF-α, IL-17, IL-23, and IL-22. These cytokines contribute to the persistent inflammatory milieu and the recruitment of immune cells to the skin, leading to characteristic psoriatic lesions. Additionally, oxidative stress, angiogenesis, and genetic predisposition further amplify the disease process (Sieminska et al. 2024).

Although numerous therapies exist including topical corticosteroids, vitamin D analogs, systemic immunosuppressants, and biologics targeting specific cytokines their use is often limited by high cost, adverse effects, immunosuppression, or loss of efficacy over time. As such, there is a growing demand for safer, cost-effective, and multi-target therapeutic alternatives (Armstrong and Read 2020; Menter et al. 2020).

Natural products have historically served as rich sources of pharmacologically active compounds. In recent years, several plant-derived agents have gained attention for their anti-inflammatory, anti-oxidant, anti-proliferative, and immunomodulatory effects relevant to psoriasis treatment (Radu et al. 2025). Compounds, such as curcumin, thymoquinone, glycyrrhizin, and boswellic acids, have shown promise in preclinical studies and early phase clinical trials of psoriasis (Fadaei et al. 2022; Agrawal et al. 2024). However, the translational potential of these agents is hampered by the lack of rigorous clinical validation, standardization, and mechanistic clarity.

The aim of this review is to bridge this gap by comprehensively analyzing the pharmacological actions, clinical relevance, and mechanistic underpinnings of natural products with anti-psoriatic activity. It further outlines the limitations in current research and proposes directions for future investigation to support the integration of phytotherapeutics into mainstream psoriasis management.

## Psoriasis pathophysiology

**Fig. 1:**
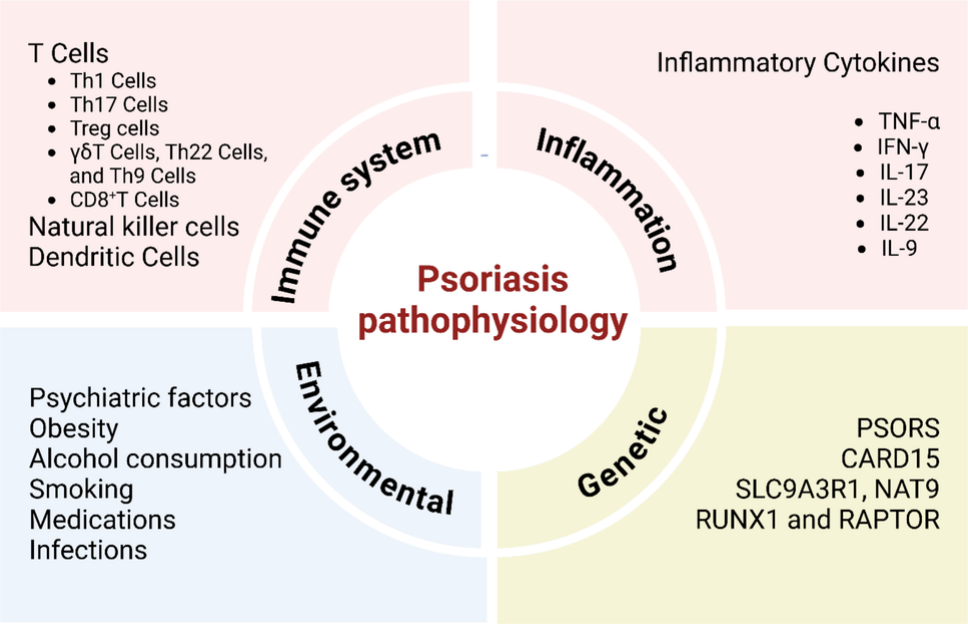
The pathophysiology of psoriasis

In psoriasis, a complex interplay between skin cells, immune cells, and different signaling molecules is set in motion by a combination of hereditary, environmental, and immunological variables. In a cellular immune response, antigen-presenting cells (APCs) in the skin release interleukin (IL)-12 and interleukin (IL-23), which activate type 1 (Th1), and type 17 (Th17) T cells. Cytokines like tumor necrosis factor (TNF) α are the main means by which these activated cells foster a chronic inflammatory state and impact processes like neo-angiogenesis, epidermal hyperproliferation, differentiation, and apoptosis. The skin symptoms that are distinctive of psoriasis are caused by this. Significant clinical improvements have been achieved with recent biologic medicines that target the immunological signaling pathways and cytokines implicated in the etiology of psoriasis. The pathophysiology of psoriasis is illustrated in Fig. 1 and Table 1**.**

**Table 1:** The pathophysiology of psoriasis

Category	Component	Role/effect	Key proteins/cytokines	Pathways/mechanisms
Immune cells	Th1 cells	Promote inflammation, keratinocyte activation	IFN-γ, IL-2	JAK/STAT, NF-κB
	Th17 cells	Major contributor to chronic inflammation	IL-17, IL-22, IL-23	IL-23/Th17 axis, RORγt
	Th22 & Th9 cells	Mediate IL-22/IL-9 secretion	IL-22, IL-9	STAT3, NF-κB
	Treg cells	Immunosuppression, balance with Th17	IL-10, TGF-β, Foxp3	IL-2 signaling
	CD8 + T cells	Target keratinocytes, perpetuate inflammation	Perforin, IFN-γ	HLA-Cw6, cytotoxic granules
	γδT cells	Rapid IL-17 production in skin	IL-17, CCR6	IL-23 responsiveness
	NKT cells	Promote Th1-type inflammation	IFN-γ, CD1d	NF-κB, MAPK
	Dendritic cells	Initiate adaptive immune response	IL-12, IL-23, TNF-α	NF-κB, MAPK, TLR signaling
Cytokines	IL-17	Drives keratinocyte proliferation/inflammation	IL-17A, IL-17F	IL-17/IL-17R axis
	IL-22	Promotes acanthosis and epidermal hyperplasia	IL-22	STAT3 pathway
	IL-23	Supports Th17 cell maintenance	IL-23 (p19 + p40)	IL-23/Th17 axis
	IFN-γ	Initiates inflammatory cascade	IFN-γ	JAK/STAT, NF-κB
	TNF-α	Central in cytokine amplification, DC activation	TNF-α	TNFR1/TNFR2, NF-κB
	IL-9	Promotes Th17 response	IL-9, IL-9R	IL-9/Th17 interaction
Environmental triggers	Psychological stress	Triggers flares, immune dysregulation	CRH, IL-6, TNF-α	HPA axis activation
	Obesity	Enhances inflammation via adipokines	Leptin, Resistin, IL-6	Adipocyte-macrophage interaction
	Alcohol	Induces keratinocyte proliferation and cytokine release	TNF-α, IL-6, Cyclin D1	ROS, immune dysregulation
	Smoking	Worsens psoriasis, activates pro-inflammatory pathways	CYP1A1, MAPK, NF-κB	Nicotinic receptor signaling
	Infections (Bacterial, Viral, Fungal)	Trigger Th17 responses, antigen mimicry	Superantigens, IFN-γ, IL-17	TLRs, complement activation, IL-23 induction
Genetic factors	PSORS1 (HLA-Cw6)	Key susceptibility locus, antigen presentation	HLA-Cw6	MHC class I presentation to CD8 + T cells
	CARD15	Activates NF-κB pathway in keratinocytes	CARD15	NF-κB, TRAF2 signaling
	EDC (LCE3B/C genes)	Barrier dysfunction	LCE3B, LCE3C	Epidermal differentiation pathways
	RAPTOR	Regulates T-cell proliferation	RAPTOR	mTOR signaling
	SLC9A3R1/NAT9	Modulate T-cell signaling	SLC9A3R1, NAT9	Signal transduction
	CARD15	Associated with psoriatic arthritis	CARD15	NF-κB via LPS response

## The role of the immune system and inflammation

Psoriasis occurs when keratinocytes proliferate excessively, and the immune system becomes hyperactive. This results in atypical behavior from several immune cells, including T cells, dendritic cells, neutrophils, and macrophages. The underlying mechanism involves a complex network of signals cytokines that facilitate communication among cells and modulate their behavior, resulting in the inflammation and symptoms characteristic with psoriasis (Deng et al. 2016).

### Role of immune cells

#### Th1 cells

It was previously believed that Th1 cells were the primary immune response mediators in psoriatic plaques. Studies have shown that IL-4 can enhance the results of illness by increasing the levels of epidermal GATA3 mRNA and protein, and at the same time decreasing the levels of IL-1β and IL-6 through transcriptional and post-transcriptional mechanisms (Aydogan et al. 2013). Improving the expression of IL-6, IL-8, TNF-α, and hBD2, differentiating Th17 and Th22 cells, and triggering the release of IL-17 and IL-22 are all IL-1β's functions. Blocking this pathway allows the Th2 cytokine IL-4 to regenerate healthy skin in psoriatic skin (Onderdijk et al. 2015). Psoriasis patients' mesenchymal stem cells (MSCs) have been shown to have an upregulation of genes that encode Th1 cytokines, but not Th2 cytokines, when compared to healthy controls (Campanati et al. 2014). The bidirectional response pattern of Th1/Th2 equilibrium clearly reveals the differences between the paradigms. Th2 cytokines (IL-4, IL-10) in erythrodermic psoriasis (EP) show higher levels than in psoriasis vulgaris (PV) (Zhang et al. 2014). Those who suffer from psoriasis and skin that is affected have increased levels of Th1 cells and IFN-γ. At the onset of the psoriatic cascade, interferon-γ has a role in promoting the production of CCL20, IL-17 + T cells, and γ-defensin2 (HBD-2), which is a protein that is both antimicrobial and chemotactic in nature. Psoriatic keratinocytes are responsible for the overexpression of this protein (Kryczek et al. 2008). It has been found that dysregulated keratinocytes, which include IL-1β and IL-18, are responsible for the release of AMP and IL-1 family cytokines, which then leads to the development of Th1 and Th17 cells (Anderson et al. 2015).

#### Th17 cells

The cytokines IL-1β, TGF-β, IL-6, and IL-23, as well as the transcription factor RORγt, regulate the development of Th17 cells. Psoriatic skin lesions contain high numbers of CD4 + T cells that produce IL-17, indicating that Th17 cells play a crucial role in the immunopathogenesis of psoriasis (Fujishima et al. 2010). To heighten the inflammatory response of keratinocytes, Th17 cells secrete IL-17, IL-12, IL-22, and IL-9. To improve the immune response in psoriasis, keratinocytes secrete chemokines, cytokines, and antimicrobial peptides such as CCL20 and CXCL1, 3 (Ottaviani et al. 2006). An IL-23/Th17 axis positive feedback loop is established when activated Th17 cells enhance the inflammatory response. According to recent animal research, the Th17 pathway plays a pivotal role in the pathogenesis of psoriasis. There is some evidence that anti-IL-23 antibody inhibition of the IL-23/Th-17 pathway may slow the course of psoriatic disease. Antibodies or knockout mice that lack IL-22 are able to inhibit IL-23-induced skin thickening (Büchau and Gallo 2007). Dysregulated IL-17 signaling and Th17 cell pathway activity facilitate persistent inflammation in psoriasis. The primary objective of ongoing therapeutic trials is to reverse this dysfunction (Deng et al. 2016).

#### Regulatory T (Treg) cells

Several stimuli, including as TGF-γ, IL-4, IFN-γ, IL-2, and IL-6, impact the polarization of regulatory T (Treg) cells. Foxp3, TGF-β, IL-10, perforin, and granzyme A are expressed by mature Treg cells. They facilitate the preservation of peripheral tolerance, mitigate chronic inflammatory disorders, and avert autoimmune diseases (Campbell and Koch 2011; Bell et al. 2015). Treg cells may inhibit other immune cells, such as Th17 cells. IL-6, essential for Treg cell development, inhibits Th17 cell activation and proliferation, hence establishing a balance between Tregs and Th17 cells. Elevated Treg cell numbers have been noted in the skin lesions of individuals with psoriasis (Dhaeze et al. 2015). In models of psoriasis, the deletion of CD18 from Tregs results in the hyperproliferation of T cells that are responsible for pathogenic immunity. In the Cd18hypo PL/J mouse model, it was found that decreased CD18 expression led to poor functioning of Treg cells as well as decreased proliferation of these cells. Using adoptive transfer, Cd18wt Tregs were transferred into Cd18hypo PL/J mice, which resolved Treg cell malfunction and improved the outcomes of the psoriasis study (Wang et al. 2008). Recently, IMQ-treated mice exhibited improved psoriasis-like skin lesions, reduced Th17 cytokine levels, and elevated Treg cell counts. CD4 + CD25 + T cells generated in vitro from the hematopoietic cells of psoriasis patients exhibit diminished regulatory functions (Kim et al. 2015).

#### γδT cells, Th22 cells, and Th9 cells

There is a strong connection between immune cells and psoriasis, specifically CD4 + T cells which create IL-9 and γά T cells which produce IL-17. The inflammatory cytokines IL-17, IL-22, and IL-9 are generated by these cells and play a role in the development of psoriasis (Cai et al. 2013). γδT cells enhance a Th17 inflammatory response by secreting IL-17 after being quickly activated by IL-23, IL-1β, or danger signals. Studies show that T-cell receptor δ-deficient (Tcrd − / −) mice have significantly decreased levels of inflammation, epidermal hyperplasia, and IL-17 (Cai et al. 2011). Furthermore, cutaneous inflammation and acanthosis caused by IL-23 or IMQ are exclusively observed in wild-type (WT) mice (Van Der Fits et al. 2009). Human investigations have identified a substantial presence of IL-17-secreting γδT cells in lesions of psoriasis patients, with dermal γδT cells serving as a major source of IL-17 and CCR6 following IL-23 activation in the skin. Vγ9Vδ2 T lymphocytes, elevated in the skin yet diminished in the peripheral blood of psoriasis patients, migrate from the bloodstream to the skin to contribute to psoriasis pathogenesis (Laggner et al. 2011). IL-22-secreting Th22 cells and IL-9-secreting Th9 cells constitute additional unique subsets of effector T cells. Research on γδT, Th22, and Th9 cells in psoriasis is anticipated to increase, enhancing comprehension of the mechanisms underlying inflammatory responses and the pathophysiology of psoriatic illness (Lowes et al. 2014).

#### CD8^+^T cells

You might be surprised to know that the PSORS1 gene is a component of the MHC. CD8 + T cells in psoriasis patients' blood often carry the HLA-Cw6 gene, the disease's most important susceptibility locus (Volarić et al. 2019). Cell surface antigens recognized by CD8 + T lymphocytes in psoriatic skin lesions included keratin and epitopes shared by Streptococcal M proteins (Johnston et al. 2004). The fact that perforin-mediated cytotoxicity of CD8 + T cells destroys psoriasis keratinocytes provides more evidence that CD8 + T cells have a role in the development of the disease. Psoriasis progresses in part because CD8 + T lymphocytes generate apoptotic keratinocytes, which initiate a destructive cycle of regenerative hyperplasia in epidermal keratinocytes (Deng et al. 2016).

#### Natural killer T cells

The innate immune system activates NKT cells promptly during the immune response because of their capacity to deliver direct cytotoxic effects and swiftly generate cytokines. This encompasses interferon-gamma (IFN-γ), which facilitates a Th1-mediated inflammatory response, and interleukin-4 (IL-4), which endorses Th2 cell development. The dysregulation or hyperactivation of NKT cells has been linked to the pathophysiology of various immune-related disorders, including multiple sclerosis, inflammatory bowel disease, and allergic contact dermatitis (Hugh and Weinberg 2018). NKT cells in psoriasis are situated within the epidermis, in close proximity to keratinocytes, indicating their role in direct antigen presentation. Furthermore, CD1d, a protein essential for NKT cell activation, is extensively increased in the epidermis of psoriatic lesions, unlike its restricted expression in terminally differentiated keratinocytes under normal circumstances. An in vitro investigation simulating psoriasis-like cytokine-driven inflammation subjected cultured CD1d-expressing keratinocytes to interferon-gamma alongside α-galactosylceramide, a glycolipid antigen from the lectin family, to evaluate immune activation (Dunphy and Gardiner 2011). Interferon-gamma (IFN-γ) was found to augment CD1d expression on keratinocytes, leading to their activation and the subsequent stimulation of NKT cells. This interaction resulted in a significant elevation of IFN-γ production by NKT cells, although IL-4 remained unmeasurable. The predominant production of IFN-γ indicates a bias toward a Th1-mediated immune response facilitated by NKT cells, which contributes to the immunopathogenesis of psoriasis (Dunphy and Gardiner 2011).

#### Dendritic cells

Psoriatic skin has a significant accumulation of several antigen-presenting cell types, especially myeloid and plasmacytoid dendritic cells, in the epidermis and dermis relative to non-lesional skin. Dermal dendritic cells are involved in eliciting inflammatory responses through the production of IL-12 and IL-23, which facilitate the activation and development of Th1 and Th17 cells, respectively (Liu and Cao 2015). The diverse biological response secretes cytokines, triggering a cascade involving keratinocytes, fibroblasts, endothelial cells, and neutrophils that generate the skin lesions typical of psoriasis (Di Cesare et al. 2009).

### The role of inflammatory cytokines

Psoriatic lesions exhibit elevated levels of TH1, TH2, and TH17 cytokines. This inflammation results in the recruitment of supplementary immune cells that generate further cytokines. Psoriatic lesions are marked by a detrimental cycle of inflammation, leading to cutaneous symptoms such as plaques (Trefzer et al. 2003). CD1d-overexpressing keratinocytes induce natural killer T cells to enhance pro-inflammatory IFN-γ production while leaving anti-inflammatory IL-4 unaffected. Antigen-presenting cells (APCs) also generate cytokines, such as IL-18, IL-23, and TNF-α, which are present in the inflammatory infiltrate of psoriatic plaques. IL-18 and IL-23 both induce TH1 cells to secrete IFN-γ; however, IL-23 additionally activates TH17 cells (Hugh and Weinberg 2018).

#### Tumor necrosis factor-alpha (TNF-α)

Both the antigen-presenting capacity of dendritic cells and the infiltration of T lymphocytes are influenced by TNF-α. In psoriasis, the levels of NF-κB are elevated, and this cytokine partially accomplishes its purpose by phosphorylating it (Goldminz et al. 2013). TNF-α acts as a regulator of the IL-23/Th17 axis and promotes IL-23 production by dendritic cells in psoriasis, suggesting that it possesses pro-inflammatory properties. By blocking IL-23 and reducing levels of Th17 effector molecules like IL-17, IL-22, chemokines, and β-defensin, Etanercept and other TNF-α inhibitors accomplish their immunological purpose. Th17 cells are directly affected by TNF-α in psoriasis, because it decreases autonomous TNF/TNFR2 signaling (Shiga et al. 2015).

#### Interferon-gamma (IFN-γ)

In the early stages of the disease, IFN-γ is more important, according to research. STAT3 is activated as a result of Janus kinase 1 (JAK2) and JAK3 being cross-phosphorylated by Interferon-gamma (IFN-γ). The activation of STAT factors plays a critical role in cell proliferation and has the ability to regulate several genes that are expressed in psoriatic skin lesions (Johnson-Huang et al. 2012). The release of cytokines (IL-23, IL-1), chemokines (CXCL10, CXCL11), and adhesion molecules from dendritic cells is induced by IFN-γ. Investigations suggest that IFN-γ, because of its favorable correlation with PASI, could function as a biomarker for the activity of psoriasis illness (Madonna et al. 2010).

#### Interlukin-17 (IL-17)

Blood and skin samples taken from patients with psoriatic arthritis show increased levels of IL-17 mRNA and protein. Th17 cells are mostly responsible for IL-17 production, but new research shows that innate immune cells, such as γδT cells, neutrophils, and mast cells, also play a role in IL-17 release in psoriasis (Isailovic et al. 2015). There are at least six homodimeric cytokines that make up the IL-17 family, and they are IL-17A through IL-17F. The two ILs that are most relevant to psoriatic disease are IL-17A and IL-17F. The antimicrobial protein REG3A, which is involved in wound healing, acts as a downstream mediator between IL-17 and keratinocytes, enhancing their proliferation and inhibiting their differentiation (Lai et al. 2012). It has been demonstrated in the past that IL-17A causes keratinocytes to produce chemokines and antimicrobial peptides. The inflammatory response in psoriasis is sustained by keratinocytes, which in turn attract Th17 cells and enhance IL-17 production, forming a positive feedback loop (Ramirez-Carrozzi et al. 2011).

#### Interlukin-23 (IL-23)

Th17 cells cannot survive or proliferate without IL-23. IL-23 accelerates the development of psoriasis by stimulating the overproduction of keratinocytes. Members of the IL-12 family include IL-23 as well as IL-12. The IL-12 P40 subunit is shared by both IL-12 and IL-23, although p35 and p19 are different subunits found in each of these two proteins (Lee et al. 2004). The main producers of interleukin-12 and interleukin-23, which are found in higher amounts in psoriatic skin, are dendritic cells and macrophages. While p35 levels do not change, new studies show that p19 and p40 mRNA are higher in lesional skin than in non-lesional skin. To summarize, IL-23, not IL-12, is the main factor that causes psoriasis. The amounts of IL-23 protein in psoriatic skin lesions are much higher than in non-lesional skin (Yawalkar et al. 2009).

#### Interlukin-22 (IL-22)

Th17 and Th22 cells mediate the effect of IL-22 on IL-23-induced keratinocyte hyperproliferation in both in vivo and in vitro settings through STAT3 signaling. Because it is exclusively expressed by epithelial cells (e.g., skin keratinocytes) and bound to its heterodimeric receptor (IL-22RA and IL-10 receptor B), IL-22 exerts its effects in tissues. People with psoriasis often have elevated IL-22 levels in their blood (Mashiko et al. 2015). The interaction between interleukin-22 and interleukin-17 inhibits keratinocyte differentiation while increasing their proliferation and motility, leading to nuclear retention within the stratum corneum, epidermal hyperplasia (acanthosis), and elongation of the epidermal rete ridges (papillomatosis) all of which are characterized by psoriatic skin plaques (Becher and Pantelyushin 2012).

#### Interlukin-9 (IL-9)

In comparison to healthy skin from control subjects, psoriatic lesional skin expressed significantly more IL-9. Research carried out by Singh et al. indicates that the skin of psoriatic mice models shows an increase in IL-9R and IL-9 expression following intradermal injection of IL-9, which initiates a Th17-related inflammatory response (Singh et al. 2013). The pro-inflammatory cytokine IL-9 secretes IFN-γ, TNF-α, IL-17, and IL-13 in psoriasis. There is IL-9 secretion by both Th9 and Th17 cells. In addition to blocking IL-9 signaling, the anti-IL-9 monoclonal antibody decreases IL-17 synthesis, which in turn diminishes Th17 functioning in another model of autoimmune illness. This data points to IL-9 as a potential mediator or target of the IL-23/Th17 pathway suppressor (Witte et al. 2014). While, near the IL-9 gene locus on chromosome 5q31.1-q33.1 is a psoriasis susceptibility gene (Deng et al. 2016).

## Environmental factors

Environmental factors can play a significant role in triggering or exacerbating psoriasis, weakening the immune system, and causing flare-ups. Common environmental triggers include psychiatric factors, cold weather, medications, infections, smoking, obesity, and excessive alcohol consumption. Identifying and managing these triggers are crucial for minimizing psoriasis episodes and improving skin health (Mahajan and Handa 2013).

### Psychiatric factors

Daily living stress is associated with the emergence of psoriasis and exacerbates its symptoms. Mental stress significantly influenced the onset, progression, and exacerbation of psoriasis. Psychological stress is recognized to influence psoriasis via immune system modulation and atypical T-cell activation (Zeng et al. 2017). Stress stimulates corticotrophin-releasing hormone (CRH), resulting in mast cell degranulation and the release of pro-inflammatory cytokines (Evers et al. 2010). The heightened expression of the CRH-R1 receptor found in psoriatic lesions may play a role in the disease's underlying processes. Pro-inflammatory cytokines frequently elevated in psoriasis, including TNF-α, IFN-α, IL-1, and IL-6, are recognized for their ability to inhibit serotonin synthesis while increasing CRH levels, potentially associating psoriasis with the onset of depressive symptoms and increased vulnerability to stress (Rousset and Halioua 2018).

### Obesity

Obesity has been demonstrated to correlate with a pro-inflammatory condition, and numerous studies indicate that excess weight may exacerbate psoriasis (Zeng et al. 2017; Yamanaka et al. 2021). The involvement of obesity in psoriasis development may be better understood by examining the mechanisms by which adipocytes and the immune system interact via several mediators, one of which being adipokines. Increased production of pro-inflammatory cytokines by macrophages and an increase in pro-inflammatory adipokines like leptin and resistin are two ways in which obesity impacts adipocytes (Wong et al. 2019), to reduce levels of adipokines that inhibit inflammation. The inflammatory cascade is amplified when adipocytes and infiltrating cells in adipose tissue interact, resulting in the generation of several inflammatory cytokines (Mizutani et al. 2020). An additional concern is hyperlipidemia, which has a role in atherogenesis through the promotion of endothelial cell adhesion molecule overexpression and the enhancement of mononuclear cell adherence due to low-density lipoprotein (Shih et al. 2020).

### Excessive alcohol consumption

Chronic alcohol use can aggravate psoriasis by impairing immune system control. Alcohol-induced immunological dysregulation results in elevated release of pro-inflammatory cytokines from diverse cell types, fostering chronic systemic inflammation and augmenting lymphocyte activation and proliferation, therefore facilitating disease progression (Farkas et al. 2003). Moreover, alcohol and acetone have been demonstrated to directly induce keratinocyte proliferation and enhance the mRNA expression of genes associated with cell growth, including α5 integrin, cyclin D1, and the keratinocyte growth factor receptor. The augmented proliferative activity is concomitant with heightened vulnerability to superficial skin infections and tissue injury (Farkas and Kemeny 2013).

### Smoking

The development of palmoplantar pustulosis and a decrease in the effectiveness of treatment have both been linked to cigarette smoking. This factor affects the levels of certain genetic markers, including HLA-Cw6, HLA-DQ*0201, and CYP1A1, and it helps activate important signaling pathways like mitogen-activated protein kinase (MAPK) and nuclear factor κB (NF-κB), which play a role in inflammatory reactions (Cheng et al. 2009). Nicotine can aggravate psoriasis by promoting neo-angiogenesis and increasing neutrophil chemotaxis. Furthermore, nicotine engages with nicotinic acetylcholine receptors (nAChRs), resulting in disturbances in standard nervous system signaling. The signaling pathways facilitated by nAChRs can influence keratinocyte activity. Additionally, several immune cells, such as T cells, B cells, thymocytes, and specific leukemic cell lines, exhibit functional nAChRs, and nicotine's interaction with these receptors can disrupt immune cell signaling and alter immunological responses (Moriwaki et al. 2015).

### Medications

Numerous drugs have been recorded as directly causing the eruption of psoriasis, alongside others that have been noted to trigger the condition (Milavec-Puretić et al. 2011) **(**Table 2**).**

**Table 2:** Drugs recorded causing or triggering the eruption of psoriasis

Drug	Mechanism
Beta-blockers	One class of medications that has been associated with a worsening of plaque psoriasis is beta-blockers, which are also called beta-adrenergic receptor antagonists. A delayed type hypersensitivity reaction, an immune-mediated response, a reduction in intraepidermal cAMP, and an increase in epidermal cell turnover are the mechanisms by which it is associated with psoriasis development (Awad et al. 2020)
Lithium	Functions directly by inhibiting cell differentiation and causing dysregulation of inflammatory cytokines, and indirectly by reducing cAMP levels. Moreover, it causes elevation of circulating neutrophil numbers and facilitation of lysosomal release from leukocytes, impacting concentrations of interferon-γ and interleukin-2 in the dermis of individuals with psoriasis (Mahajan and Handa 2013)
Antimalarial drugs: chloroquine and hydroxychloroquine	Synthetic antimalarial medications chloroquine and hydroxychloroquine are linked to exacerbations of plaque-type psoriasis. Numerous accounts exist of psoriasis exacerbation in persons undergoing antimalarial prophylaxis. Additionally, instances of psoriasis exacerbation have been documented with the use of hydroxychloroquine as an immunomodulatory therapy for lichen planopilaris and psoriatic arthritis. These medications may induce psoriasis by blocking the enzyme transglutaminase (Gravani et al. 2014)
Imiquimod	The TLR7 agonist imiquimod is employed as a topical therapy for multiple reasons, including (pre)-malignant skin lesions. Imiquimod therapy may exacerbate psoriasis in adults with an existing diagnosis of the condition (Gilliet et al. 2004)
NSAIDs	Accumulate leukotrienes, which may exacerbate psoriasis, by inhibiting the cyclo-oxygenase pathway (Basavaraj et al. 2010)
Tetracyclines	Tetracyclines likely induce psoriasis by decreasing intracellular cAMP or by interacting with arachidonic acid and its metabolites. Tetracyclines accumulate in greater amounts in psoriatic lesions compared to clinically unaffected skin. It is widely recognized that they function as photosensitizing agents. Patients predisposed to psoriasis may experience a Koebner reaction due to the photosensitizing effects of tetracyclines (Tsai and Tsai 2019)

### Infections

Recent research suggests that infections serve as environmental catalysts for psoriasis and contribute to its chronic nature, particularly highlighting a significant correlation between the emergence of guttate psoriasis and acute streptococcal infections. Various pathogens can provoke immune cells to generate inflammatory cytokines, thereby initiating or exacerbating psoriasis. Besides bacterial infections, viral and fungal infections have been significantly associated with the onset or worsening of psoriasis (Zhou and Yao 2022).

#### Bacterial infection

One of the basic mechanisms is superantigens. A large number of superantigens are usually produced by bacterial isolates. To connect antigen-presenting cells to T-helper cell receptors, superantigens cling to the outside surface of MHC class II proteins. After then, T cells become active, which causes them to multiply and produce cytokines like IFN-γ (Yan et al. 2017). Overstimulation, on the other hand, causes energy-related T-cell failure and dysfunction. In addition, superantigens increase the synthesis of the skin-homing receptor, cutaneous lymphocyte antigen, by T cells, which helps with Th17-dominated responses (Hedin et al. 2021). Peptidoglycan (PG) is involved in a distinct process. Dendritic cells and macrophages can take up peptidoglycan from bacteria in the intestines or tonsils, and then, dendritic cells and macrophages can go to the skin to provide peptidoglycan peptides to clones of Th1 cells that are specific to antigens. Keratinocytes are influenced by cytokines, especially IFN-γ. In the end, the epidermal layer becomes too proliferant and fails to differentiate adequately (Zhou and Yao 2022).

#### Viral infection

Researches indicate the significant role of viral infections in the pathophysiology of psoriasis, proposing that deregulation of host antiviral immune responses initiates the inflammatory skin conditions associated with the disease's development (Zhu et al. 2017). Viruses (namely, HIV, HCV, and HPV) play a role in psoriasis. Cell proliferation occurs through PKC-dependent or -independent pathways, facilitated by the tat gene, which in turn causes HIV replication. IFN-γ, cathelicidin, and TLR9 are all genes that HCV boosts in order to make inflammation easier. When anti-HPV antibodies bind to viral proteins, it triggers the activation of complement and the development of Munro's microabscesses (Teng et al. 2021).

#### Fungal infection

Some clinical studies suggest a possible connection between fungus and psoriasis. Some species of Malassezia yeasts, such as those associated with guttate and scalp psoriasis, have been associated to different forms of the skin condition (Aydogan et al. 2013). In addition, Candida albicans infection has been linked to newborn diaper psoriasis (Bonifaz et al. 2016). Based on these findings, it was postulated that psoriasis develops when fungus, including the scalp-found Malassezia ovalis, trigger the alternative complement pathway. After 4 weeks of treating four cases of scalp psoriasis with ketoconazole, the lesions improved and the researchers found no evidence of fungal growth on the scales (Zhou and Yao 2022). Several epidemiological studies have shown that Candida albicans and Malassezia spp. are the most common types of fungi associated with psoriasis. According to the characteristics of these fungi, the majority of fungal infection sites in psoriasis patients are found in skin creases and places where sebum accumulates (Vijaya Chandra et al. 2021). By affecting the development of specific types of T cells, malassezia can bring on or worsen psoriasis in those who already have it. Malassezia glabrata's hydrophobic components cause human epidermal keratinocytes to produce inflammatory proteins, which in turn cause peripheral blood Th1-biased differentiation of Th cell subsets. The study found that the fungus Malassezia furfur affects cell migration and proliferation by increasing the expression of transforming growth factor-β1, integrin chains, and HSP70 in human keratinocytes. The subjects included both positive and negative skin biopsies from psoriasis patients (Baroni et al. 2004). The Candida albicans surface proteins behave as superantigens, which trigger the activation of certain Vβ family T lymphocytes even in the absence of antigen presentation, leading to an excess of pro-inflammatory cytokines (Javad et al. 2015). Interleukin-23 (IL-23) facilitates the multiplication and survival of Th17 cells, which are essential for combating Candida albicans infections. Th17 cells produce IL-17, a cytokine that attracts neutrophils to areas of infection and promotes the synthesis of antimicrobial peptides, therefore aiding in the elimination of Candida species. Nonetheless, despite the protective function of IL-17, individuals receiving monoclonal antibody therapy aimed at IL-17A or its receptor for psoriasis have exhibited a heightened occurrence of Candida infections. This indicates that although IL-17 inhibitors effectively control psoriasis, they may compromise the body's defenses against fungal infections (Furue et al. 2020).

## Genetic factors

Psoriasis is an autoimmune disorder affecting genetically predisposed individuals. Due to the variability in skin phenotypes, extra-cutaneous symptoms, and disease progression among patients, it is posited that many genes arising from linkage disequilibrium contribute to the pathogenesis of psoriasis (Hugh and Weinberg 2018).

### Psoriasis susceptibility (PSORS)

Linkage research discovered nine distinct areas, termed Psoriasis Susceptibility (PSORS) 1–9 that are believed to influence disease susceptibility. Among these, only PSORS1 was thoroughly verified across all assessed cohorts (Consortium 2003). This prompted researchers to discern a substantial genetic factor contributing to the condition. Weaker linkage signals were detected at the PSORS2 and PSORS4 loci in several datasets, indicating that these regions are authentic susceptibility loci. Nonetheless, the correlations with other PSORS regions (PSORS3, PSORS5, PSORS6, PSORS7, PSORS8, and PSORS9) could not be corroborated in independent investigations (Capon 2017). Decades of genome-wide linkage scans have confirmed that PSORS1 is the most significant susceptibility locus identified through familial linkage studies, accounting for up to 50% of the genetic contribution to psoriasis (Rahman and Elder 2005). The PSORS1 gene is located inside the major histocompatibility complex (MHC) on chromosome 6p21. Following the definitive validation of connectivity to this region, fine-mapping studies were conducted by multiple research groups. These investigations established a consensus minimal interval of 150 kb that encompasses the MHC class I area and includes nine genes (Lesueur et al. 2007). Among these, three genes (HLA-C, CCHCR1, and CDSN) exhibited considerable polymorphism and had coding variations significantly correlated with psoriasis (Capon et al. 2002). HLA-C encodes an MHC class I receptor that contributes to immunological responses by presenting antigens to CD8 + T cells. Consequently, it is a highly credible candidate gene, particularly since serological tests conducted in the mid-1970s found a correlation between psoriasis and the HLA-Cw6 allele (Chen and Tsai 2018). The function of CCHCR1 remains little comprehended, whereas CDSN encodes a keratinocyte protein implicated in skin desquamation, a process recognized to be disrupted in psoriasis (Jonca et al. 2011). Although the preceding data suggested a role for CDSN variation in psoriasis etiology, further investigations indicate that the correlation with CDSN alleles is likely secondary, indicating linkage disequilibrium with HLA-Cw6 (Strange et al. 2010). Recent studies suggest that HLA-Cw6 may contribute to psoriasis molecular pathogenesis by binding to psoriasis auto-antigens. HLA-Cw6 can present specific melanocyte auto-antigens to CD8 + T cells and has a high affinity for LL-37, a T-cell autoantigen in psoriasis. As T-cell infiltrates are polyclonal, more molecules may be recognized by HLA-Cw6 (Mabuchi and Hirayama 2017).

The PSORS2 region was first found via linkage analysis of a multi-generational North American pedigree in which psoriasis was transmitted as an autosomal dominant trait (Capon 2017). The underlying genetic flaw responsible for disease inheritance in these two families was found to be mutations in the CARD14 gene, according to next-generation sequencing. Two diseases that share symptoms with plaque psoriasis pityriasis rubra pilaris and generalized pustular psoriasis—have been associated with these CARD14 mutations (Fuchs-Telem et al. 2012). The adaptor protein encoded by CARD14 is mostly present in keratinocytes and is essential for TRAF2-mediated NF-κB signaling. Characterized mutations in CARD14 cause gain-of-function consequences, which result in increased production of pro-inflammatory cytokines and sustained activation of NF-κB (Berki et al. 2015). Common CARD14 SNPs associated with psoriasis in case–control studies have been discovered in genome-wide association analyses. Multiplex pedigrees often include rare, highly penetrant mutations in CARD14, as well as common, less significant susceptibility alleles. Because CARD14 mutations are found in a small number of psoriasis families, there are still genes that have not been found that are linked to monogenic forms of the disease (Tsoi et al. 2012).

A highly conserved genomic area comprising over 60 genes involved in terminal keratinocyte differentiation is the Epidermal Differentiation Cluster (EDC), which is part of the PSORS4 locus on chromosome 1q21. Two EDC genes, LCE3B and LCE3C, are closely linked to psoriasis, according to recent research. These genes encode proteins for the late cornified envelope. These findings point to the possibility that these deletions, by interfering with the skin's protective barrier function, exacerbate the condition (De Cid et al. 2009).

### SLC9A3R1, NAT9, RUNX1, and RAPTOR

Genomic analyses have identified more susceptibility loci for psoriasis on chromosomes 1q21, 3q21, 4q32-35, 16q12, and 17q25. Recent mapping localized two sites on chromosome 17q, revealing a separation of 6 megabase pairs, thus demonstrating distinct linkage factors. The genes SLC9A3R1 and NAT9 are located in the first region, whereas RAPTOR is identified in the second region (Hugh and Weinberg 2018).

SLC9A3R1 and NAT9 are regulators of signal transduction, the immunologic synapse, and T-cell proliferation (Krueger and Bowcock 2005). There is a common mutation in the population that is located near SLC9A3R1 and NAT9. This mutation is likely caused by the deletion of an RUNX1 binding site at the 1.2 kb region 39 of both genes. On the other hand, it has a small impact on psoriasis vulnerability. This is in line with the commonly held belief that psoriasis susceptibility polymorphisms are prevalent in the community but have little effect on individuals, indicating the presence of other variables that increase vulnerability to the illness. In addition to its role in hematopoietic cell formation, the transcription factor RUNX1 may affect the immune response in keratinocytes, peripheral areas, synovium, bone marrow, and the thymus (Helms et al. 2003).

RAPTOR participates in T-cell functionality and development pathways. This example suggests that modifications to regulatory genes, including those not yet identified, may augment T-cell proliferation and inflammation observed in psoriasis. RAPTOR is located in the psoriasis linkage area on chromosome 17q25, initially identified in a big family with several psoriasis cases (Tokuhiro et al. 2003). Several members of this family also manifested psoriatic arthritis (PsA) at a young age. Therefore, it is plausible to conjecture that a regulatory variation in RAPTOR predisposes individuals to both psoriasis and psoriatic arthritis (PsA) (Filer et al. 2009).

### CARD15

Additional regions that may increase susceptibility to PsA have been identified by two genome-wide scans. One of these is on chromosome 16q, which happens to be where a psoriasis locus is located. On chromosome 15q, you will find the other one (Krueger and Bowcock 2005). Polymorphisms within CARD15 on chromosome 16q predispose individuals to PsA, according to recent findings on a cohort of PsA patients from Newfoundland. These polymorphisms were found in 28% of patients compared to 12% of controls. In response to lipopolysaccharides produced by bacteria, CARD15 may activate nuclear factor kB (NFkB) (Lascorz et al. 2005).

## Interconnected mechanisms in the pathophysiology of psoriasis

Psoriasis is a chronic, immune-mediated, inflammatory skin disorder marked by abnormal keratinocyte proliferation and differentiation, sustained by a dysregulated immune response (Lowes et al. 2014). Its pathogenesis is not attributable to a single factor but is the result of a complex and dynamic interaction among genetic predispositions, environmental stimuli, and immune dysregulation, each of which contributes to initiating and perpetuating the disease cascade. This multifaceted process is best understood not as a linear sequence of events but as a self-perpetuating immunopathological circuit, where feedback between the immune system and epidermal cells amplifies the inflammatory response (Baliwag et al. 2015).

At the onset, environmental triggers including infections (e.g., streptococcal pharyngitis), trauma (Koebner phenomenon), psychological stress, smoking, or medications activate APCs such as dendritic cells and macrophages within the dermis (Hugh and Weinberg 2018; Zhou and Yao 2022). These innate immune cells recognize PAMPs or DAMPs, leading to the activation of TLRs and subsequent production of key pro-inflammatory cytokines, including IL-12 and IL-23. These cytokines serve as pivotal molecular switches that initiate the differentiation of naïve T cells into effector subtypes Th1 and Th17, respectively (de Alcantara et al. 2021).

The Th17 axis is now recognized as a central driver of psoriatic inflammation. Activated Th17 cells secrete IL-17A, IL-17F, and IL-22, all of which have potent effects on keratinocytes, stimulating them to produce inflammatory mediators (e.g., IL-1β, IL-6, TNF-α), chemokines (e.g., CCL20, CXCL1-3), and antimicrobial peptides (e.g., β-defensins and S100 proteins). These molecules, in turn, amplify immune cell recruitment and sustain local inflammation, thereby maintaining the chronicity of the disease (Krueger and Brunner 2018).

Importantly, keratinocytes are not merely passive recipients of immune signals but actively shape the inflammatory milieu. Upon stimulation by Th17 and Th1 cytokines, keratinocytes activate intracellular signaling pathways particularly NF-κB, MAPK, and STAT3 which regulate gene transcription involved in inflammation, proliferation, and cell survival. As a result, keratinocytes undergo hyperproliferation, leading to acanthosis (epidermal thickening), and exhibit impaired terminal differentiation, culminating in the formation of scaly plaques characteristic of psoriasis (Xu et al. 2019). The release of vascular endothelial growth factor (VEGF) by keratinocytes and infiltrating immune cells also contributes to neo-angiogenesis, another hallmark of lesional skin (Marina et al. 2015).

Overlaying this immunological network is a strong genetic predisposition, with the most well-established risk locus being PSORS1 within the MHC region on chromosome 6p21. The HLA-Cw6 allele, in particular, enhances the presentation of auto-antigens to CD8 + cytotoxic T lymphocytes, promoting a cytotoxic response against keratinocytes. Additional susceptibility genes such as CARD14, which modulates NF-κB signaling in keratinocytes, and deletions within the EDC, which affect barrier function and cornification, further predispose individuals to exaggerated immune responses and impaired epidermal homeostasis (Capon 2017).

The interconnection between immune and non-immune cells creates a vicious cycle: activated immune cells trigger keratinocyte activation; in turn, keratinocytes amplify and sustain the inflammatory environment by producing cytokines and chemokines that further recruit immune cells. Moreover, the cross-talk between dendritic cells, T cells, and keratinocytes ensures the persistence of inflammation, even in the absence of a continued external trigger. This feed-forward loop, potentiated by genetic and environmental influences, is the defining feature of the psoriatic disease process (Hugh and Weinberg 2018).

In summary, the pathogenesis of psoriasis reflects an orchestrated interaction between genetic susceptibilities, environmental factors, immune cell subsets (notably Th17, Th1, and regulatory T cells), keratinocytes, and a network of pro-inflammatory mediators. This multidirectional interplay, encapsulated in Fig. 1, provides a comprehensive framework that not only explains the clinical manifestations of psoriasis but also underpins current and emerging targeted therapies aimed at interrupting specific nodes of the inflammatory circuit most notably the IL-23/IL-17 axis, TNF-α, and JAK/STAT pathways.

## Methodology for selecting natural products with anti-psoriatic activity

To identify natural products with potential benefits in treating psoriasis, we performed a focused literature search using databases, such as PubMed, Scopus, and Google Scholar. The search included studies published from 2010 to 2024, using keywords like “psoriasis,” “natural products,” “anti-inflammatory,” “anti-oxidant,” and “immune modulation.”

We included studies that:

- Reported experimental data from clinical studies.
- Investigated the effects of natural or plant-derived products on psoriasis-related mechanisms.
- Described the product’s impact on inflammation, oxidative stress, keratinocyte proliferation, or immune response.

We excluded:

- Review articles without original data.
- Studies unrelated to psoriasis or lacking mechanistic relevance.

The selected natural products were organized based on their main mechanisms of action, including anti-inflammatory, anti-oxidant, anti-proliferative, or immunomodulatory effects. Priority was given to agents supported by clinically relevant to psoriasis.

## Natural products with anti-psoriatic activity

The development of novel, low-risk treatments for psoriasis is highly desirable, given the potential side effects associated with conventional therapies. Herbal drugs, widely perceived as safer alternatives, have garnered significant attention due to their structural diversity and multifaceted mechanisms of action, a characteristic rarely found in synthetic molecules. These characteristics contribute to their appeal as promising agents for psoriasis management.

Herbal treatments may offer a cost-effective and safer therapeutic option, potentially reducing side effects and toxicity compared to the traditional therapies. Consequently, researchers continue to explore novel herbal products and their active constituents as potential psoriasis treatments. Several herbal compounds have shown promise in this context.

### Nigella sativa

Its active compound, thymoquinone, exhibits anti-oxidant properties, which could be beneficial in treating psoriasis by addressing the oxidant-anti-oxidant imbalance regularly present in psoriasis. This makes it an excellent candidate for complementary therapy with methotrexate, a standard psoriasis treatment known to induce oxidative stress. This anti-oxidant activity of nigella sativa was discovered through reversing the oxidative stress of MTX by lowering the rise of serum malondialdehyde (sMDA) associated with MTX therapy (Ahmed Jawad et al. 2014; Ahmed et al. 2014; Rida and Gladman 2020).

### Licorice root (glycyrrhizin)

This compound acts as an anti-oxidant, inhibiting reactive oxygen species and cytokine secretion. It has been shown to ameliorate psoriasis-like skin lesions and protect against liver abnormalities, making it a suitable complementary therapy for hepatotoxic psoriasis treatments like acitretin (Wu and Zhang 2015; Xu et al. 2020; Yu et al. 2020).

### Turmeric (curcumin)

Known for its potent anti-inflammatory activity, curcumin inhibits various inflammatory mediators involved in psoriasis pathogenesis. Its ability to modulate T-cell activity further supports its potential as a therapeutic agent (Sarafian et al. 2015; Bahraini et al. 2018) (Gunasekaran Shathirapathiy et al. 2015).

### Aloe vera

Exhibiting anti-inflammatory properties, aloe vera inhibits the arachidonic acid pathway and reduces TNF-α levels, contributing to its potential in psoriasis treatment (Choonhakarn et al. 2010).

### Frankincense (boswellia)

Its active derivatives, such as boswellic acids, reduce inflammatory mediators like leukotriene synthesis via 5-lipoxygenase (5-LO) and suppress T-cell and keratinocyte hyperproliferation, making it a promising candidate for managing psoriasis symptoms (Fadaei et al. 2022).

### Pumpkin oil

Being rich in nutrients and having skin nourishing and moisturizing characteristics, pumpkin oil promotes skin barrier function, which is often compromised in psoriatic patients, thereby offering a supportive role in treatment (Kolahdooz et al. 2018). Overall, these herbal supplements demonstrate significant potential as safer and more cost-effective alternatives or complements to conventional psoriasis therapies. Supportive clinical trial is included in Table 3.

**Table 3:** Clinical trials of natural products with anti-psoriatic activity

	Herbal plant	Sample	Doses	Parameters	Outcome results	Conclusion	Reference
1	*Nigella sativa* 500mg grinded seed	60 individuals with plaque, palmoplantar, or guttate psoriasis (moderate-sever)- 3 months	**Gp1:** 20% w/w NS oint. twice day + 500 mg NS tab. TID **Gp2:** 15 mg of MTX tab. once weekly, **Gp3:** MTX + NS (topically + orally)		**NS:** 60% marked response + 33% relapses **MTX:** 80% marked response + 56% relapses **Combination:** 90% marked response + 27% relapses	NS enhanced MTX efficacy. topical & oral NS effective & safe for moderate-to-severe psoriasis	(Ahmed et al. 2014)
2	*Nigella sativa*	60 patients divided into 3 groups	Gp 1: 10% w/w ointment of (NS), Gp 2: 500 mg tablet once a day Gp 3: both ointment & oral tablet	1. PASI score 2. Serum malondialdehyde, MDA	**Gp1:** complete clearance & excellent or good responses in 65% of patients, with 31% relapse rate **Gp2:** good responses in 50%, with 50% relapse rate **Gp3:** complete lesion clearance & excellent or good responses in 85%, with 18% relapse rate	NS exhibits anti-psoriatic properties, with the most effective results achieved through a combination of its ointment and oral dosage forms	(Ahmed Jawad et al. 2014)
3	*N. sativa* (black seed) cold-pressed 100% oil marketed OTC topically	56-year-old man who was diagnosed with PsA	100% oil marketed OTC topically & orally around 2 teaspoons (10 g) of this extract containing 400 mg of thymoquinone		Significant skin improvement within one week of topical treatment. Relief from arthralgias and complete elimination of knee pain after 3 days of oral treatment		(Rida and Gladman 2020)
4	Durr Derma = black cumin, olive oil, tea tree oil, cocoa butter, vitamin A & B_12_	12 patients (8 males and 4 Females)	Twice-daily treatment for 3 months	PASI score, BSA and DLQI were assessed	(PASI reduction of > 75%) in 10 of the 12 treated patients (83%)	The new preparation appears to be a promising complementary therapy for managing psoriasis	(Michalsen et al. 2016)
5	The resin of *Boswellia* spp*.*, pulp of *Cucurbita pepo* L., and roots of *Glycyrrhiza glabra*,	For 4 weeks, 108 volunteers in 2 gps applied boswellia cream or vehicle cream twice daily	The affected areas were to be covered with 1g of topical cream, which is equivalent to two Fingertip Units	DLQI & PASI as basic outcomes. BSA, PGA, pruritus severity score, & patient satisfaction as secondary outcomes	Herbal treatment relieved symptoms & improved life quality of mild-to-severe plaque psoriatic patients	This herbal cream provides significant relief from the signs and symptoms of psoriasis	(Fadaei et al. 2022)
6	Glycyrrhizin	9 patients of GPP (generalized pustular psoriasis) with LTA (Liver test abnormalities)	Acitretin (0.5 mg/kg/day orally) + glycyrrhizin (80 mg intravenously). Then maintained on tapered doses of acitretin (20–30 mg orally) and glycyrrhizin (150 mg orally)		In 2 weeks, all patients experienced at least a 77% improvement in the severity score, with significant decrease in liver enzymes. Improvement remained stable for the 12-month follow-up period	The combination of acitretin and glycyrrhizin is an effective and safe treatment option for patients with GPP who have LTA	(Yu et al. 2020)
7	Glycyrrhizin	100 patients divided to acitretin (Aci) group & (Aci + Glyc) group	Aci gp: (3 × 10 mg per day) for 8 weeks (Aci + Glyc): received an additional 3 × 75 mg of compound glycyrrhizin daily	The percentage of Th17 cells in peripheral blood and serum concentrations of IL-6, IL-17, IL-22, and TGF-β, along with PASI scores, were assessed both before and after	**(Aci + glyc)** showed more reduction in Th17, IL-6, IL-17, IL-22, and TGF-β than **Aci gp**. With higher efficacy rates 90.0% vs 76.0%	Combining glycyrrhizin with acitretin significantly enhances psoriasis treatment by suppressing Th17 cell differentiation	(Wu and Zhang 2015)
8	Oral compound glycyrrhizin (OCG) plus topical corticosteroid (TCS) (i.e., 0.1% mometasone furoate ointment)	Out of 189 patients, 92 or 93.9% were in the OCG + TCS group, while 97 or 96.0% were in the placebo + TCS group	For 28 days, patients took 75 mg of OCG or placebo pills three times a day with a daily dose of topical corticosteroids (TCS)	EASI, IGA score, and VAS score for itching were all part of the standardized examinations	OCG gp reduced IGA scores compared to the placebo & lower EASI scores compared (− 3.41 vs. − 2.71) and higher response rates: 96.8% vs. 87.9% (EASI-50) &47.9% vs. 21.2%. (EASI-75)	This provides more evidence that OCG may be useful as a nonsteroidal addition to TCS for the management of persistent dermatitis	(Xu et al. 2020)
9	Hydro alcoholic turmeric extract topically	34 patients (mild- moderate) plaque psoriasis	Turmeric microemulgel twice daily for 9 weeks	Assessments included PASI score, photographic documentation, DLQI questionnaire to evaluate quality of life	Progressive reduction in thickness, erythema and pruritus, leading to acceptable improvement in many cases, & significant resolution of psoriatic lesions in some cases	Treatment resolved & improved lesions compared to baseline & placebo. It was well tolerated, with only a minimal number of side effects	(Sarafian et al. 2015)
10	Turmeric (*Curcuma longa* L.) washout topical treatment	30 patients (mild- moderate) scalp psoriasis	Twice a day for 9 weeks	The study assessed DLQI questionnaire, PASI scores, and medical photographs taken before, during, and after treatment	Turmeric improves erythema, scaling, and induration of lesions (PASI score), and quality of life outcomes for participants (P < 0.05)	This treatment is safe & effective for scalp psoriasis with no adverse effects even with prolonged use	(Bahraini et al. 2018)
11	Turmeric (*Curcuma longa* L.) starch-fortified turmeric baths (SFTBs)	60 psoriatic participants randomized into 2 gps	Apply the paste to the body for 40 min daily over a 10-day period	PASI scores were evaluated at baseline and post-intervention (day 10)	Both groups demonstrated measurable progress across the study period	SFTBs enhance both BSA and PSI, offering a safe, cost-effective therapeutic for psoriasis	(Gunasekaran Shathirapathiy et al. 2015)
12	1% curcumin topical gel	16 patients	Applied twice daily as a thin layer, with evaluations conducted for 2 weeks and repeated after 2 weeks	DLQI and QOL-Itchy Skin Index were evaluated. Statistical methods (as paired t-tests, chi-square tests, and Mann–Whitney tests) are employed for inferential analysis	Significant improvement in QOL-Itchy scores which confirm the medication's efficacy	Topical curcumin (cost-effective treatment with minimal side effects) may serve as a valuable therapeutic option for psoriasis	(Yaghoobi et al. 2018)
13	Turmeric	12-week open-label clinical trial of 20 participants (mild- moderate) plaque psoriasis	A daily OTC turmeric and salicylic acid gel, paired with a shea butter and salicylic acid exfoliating moisturizer	Clinical evaluations at baseline & Weeks 4, 8, and 12 using a 5-point severity scale (0 = absent to 4 = severe) changes in erythema, desquamation, induration, and IGA	Improvements in weeks 4, 8, and 12 By Week 12, reductions reached for erythema (48%), desquamation (46%), induration (51%), and IGA scores (48%). No irritation or edema was observed	Demonstrated strong safety and effectiveness in plaque psoriasis following 12 weeks of daily application	(Draelos 2022)
14	Turmeric	59 patients randomized into 2 gps: a turmeric (n = 30) and a clobetasol (n = 29) gp	Clobetasol 0.05% and turmeric 1% creams twice daily for 4 weeks	-Average time to reach clinical remission -Average PASI scores at each visit -Average pruritus scores at each visit -Frequency of adverse events	No significant differences between 2 gps (Turmeric vs Clobetasol) in: Clinical remission rates: 25% vs 35%, time for remission: 4 vs 3.38 weeks, PASI scores: 8.77 vs 7.26, Pruritus scores: 6.9 vs 5.83	Topical 1% turmeric cream demonstrated non-inferior efficacy and comparable tolerability compared to standard 0.05% clobetasol cream for plaque psoriasis (mild- moderate)	(Guevara et al. 2019)
15	Oral curcumin 2 g per day (Meriva, a commercially available lecithin-based curcumin delivery system	63 patients (mild- moderate) psoriasis, for 12 weeks	1st gp: topical steroids + oral curcumin, 2nd gp: topical steroids alone	Serum levels of IL-17 and IL-22 were performed. (a cytokine implicated in psoriatic inflammation)	Significant improvement more substantial in combination gp compared to topical gp. with significant decrease in IL-22 levels	Curcumin enhanced conventional therapies through lowering IL-22 levels. supporting its role as complementary therapy	(Antiga et al. 2015)
16	Topical *aloe vera* (AV) & 0.1% triamcinolone acetonide (TA)	80 patients randomly received AV or 0.1% TA cream	1st gp (AV cream) & 2nd gp (0.1% TA cream) topically twice daily for 8 weeks	PASI and DLQI used to assess psoriasis and its impact on patients' lives	**-AV gp:** PASI scores fell (from 11.6 to 3.9), and DLQI scores dropped (from 8.6 to 2.5) **-TA gp:** PASI scores decreased (from 10.9 to 4.3), and DLQI scores improved (from 8.1 to 2.3)	Clinical symptom management may favor AV cream over 0.1% TA cream, yet both achieve similar patient-reported quality of life improvements in cases of mild-to-moderate severity	(Choonhakarn et al. 2010)
17	Dermavit cream = (*Achillea millefolium* L.), (*Calendula officinalis* L.), (*Salvia officinalis* L.),	30 patients for 2 months		Erythema (skin redness), cutaneous thickening, pruritus (itching), and desquamation (skin peeling)	90% of patients had a reduction in skin redness, thickening, and scaling. The cream hydrates, softens the skin, and promoted regeneration	The effectiveness of Dermavit cream in alleviating psoriasis symptoms can be confirmed	(Hamzic et al. 2022)
18	Topical chamomile-pumpkin oleogel (ChP)	37 patients (mild- moderate) plaque psoriasis	Twice daily for 4 weeks	PSI and PGA scale were assessed at the start and after treatment	**ChP gp**: reduction in PSI scores (4.09), 35% marked-to-complete improvement **placebo:** minimal change (0.48) with no improvement	Topical application of ChP may offer a safe and effective complementary approach for managing psoriasis plaques	(Kolahdooz et al. 2018)

*NA* Not all clinical trials provided power analysis or full statistical data

## Pharmacokinetics of key components of the natural products.

- C**urcumin** exhibits poor oral bioavailability due to low aqueous solubility, rapid metabolism, and systemic elimination. However, lecithin-based formulations such as Meriva® have demonstrated enhanced absorption and bioavailability. In a human study, Meriva® significantly improved curcumin's plasma levels compared to standard preparations (Anand et al. 2007; Antiga et al. 2015).
- **Thymoquinone**, the major active compound in *Nigella sativa*, displays rapid absorption and distribution with a short plasma half-life (approximately 1 h). Lipid-based formulations or nanoemulsions can significantly enhance its systemic bioavailability (Alkharfy et al. 2015; Verma et al. 2022).
- **Glycyrrhizin** is metabolized in the intestine to glycyrrhetinic acid and undergoes enterohepatic circulation. It has moderate bioavailability but a relatively long half-life (~ 6–10 h), which supports its sustained anti-inflammatory effects. Bioavailability varies depending on the route of administration and the glycyrrhizin form used (Krähenbühl et al. 1994; Kim et al. 2000)
- **Boswellic acids**, particularly 11-keto-β-boswellic acid (KBA) and acetyl-11-keto-β-boswellic acid (AKBA), have low oral bioavailability due to poor water solubility and first-pass metabolism. Formulations with lipid-based or phospholipid carriers improve absorption (Sharma et al. 2010; Abdel-Tawab et al. 2011).

## Mechanism of action of natural anti-psoriatic agents

Natural products play a significant role in the management of psoriasis through their anti-inflammatory, antioxidative stress, anti-hyperproliferative, and anti-angiogenic properties. Table 4**.**

**Table 4:** Summary of the mechanism of action of natural anti-psoriatic agents

Mechanism	Compound	Source	Target(s) / Pathways	Reported Effect in Psoriasis	Type of study	Reference
Anti-inflammatory	Curcumin	*Curcuma longa*	NF-κB, MAPK, IL-17, TNF-α, IL-22	Reduces pro-inflammatory cytokines and keratinocyte proliferation	Clinical + Preclinical	(Antiga et al. 2015; Zhang et al. 2022)
	Glycyrrhizin	*Glycyrrhiza glabra* (Licorice)	NF-κB, MAPK, TNF-α	Ameliorates psoriatic lesions via inflammatory inhibition	Clinical + Preclinical	(Xiong et al. 2015)
	Rosmarinic acid	*Rosmarinus officinalis*	IL-17A, IL-23	Reduces redness, scaling, and inflammation	Preclinical	(Ho et al. 2022)
	Rhododendrin	*Rhododendron* spp.	TLR-7, NF-κB, MAPK	Downregulates cytokine production, reduces inflammation	Preclinical	(Jeon et al. 2017)
	Boswellic acids	*Boswellia serrata*	IL-12, IL-23, dendritic cells	Suppresses cytokine release and immune cell activation	Clinical + Preclinical	(Halim et al. 2020)
	Resveratrol	Grapes, berries, peanuts	NF-κB, IL-17	Reduces cytokines, keratinocyte proliferation	Preclinical	(Kjær et al. 2015)
Antioxidant	EGCG (Green tea)	*Camellia sinensis*	SOD, CAT, MDA, ROS	Scavenges ROS, enhances anti-oxidant enzyme activity, reduces inflammation	Preclinical	(Zhang et al. 2016, 2024)
	Quercetin	Apples, onions	SOD, CAT, GSH, MDA	Boosts anti-oxidant defenses, reduces lipid peroxidation	Preclinical	(Chen et al. 2017)
	Carotenoids	Cyanobacteria	ROS	ROS scavenging; anti-inflammatory topical potential	Preclinical	(Lopes et al. 2020)
	Salidroside	*Rhodiola rosea*	SIRT1, NF-κB, MAPK	Reduces oxidative stress and inflammation	Preclinical	(Xu et al. 2019)
Anti-proliferative	Indirubin	*Indigo naturalis*	CDKs, keratinocytes	Inhibits keratinocyte proliferation via cell cycle arrest	Preclinical	(Gamret et al. 2018; He et al. 2022)
	Delphinidin	Berries, grapes	PI3K/Akt/mTOR	Suppresses keratinocyte proliferation	Preclinical	(Chamcheu et al. 2017)
	*Mahonia aquifolium*	Oregon grape	NF-κB, IL-8, keratinocytes	Reduces cytokines and keratinocyte proliferation	Clinical + Preclinical	(Zahir et al. 2013; Sarkar et al. 2023)
	*Artemisia capillaris*	Artemisia spp.	Apoptosis pathways	Induces apoptosis in hyperproliferative keratinocytes	Preclinical	(Lee et al. 2018)
Anti-angiogenic	Qingre Huoxue Decoction	Traditional formula	VEGF, HIF-1α, Flt-1	Decreases vascular permeability and angiogenesis	Preclinical	(Li et al. 2021)
	PSORI-CM02	Herbal formula (5 components)	VEGF, HIF-1α, HUVECs	Inhibits angiogenesis via oxidative stress and inflammation pathways	Preclinical	(Lu et al. 2021)
	Rottlerin	*Mallotus philippensis*	HUVECs	Inhibits capillary structure formation	Preclinical	(Min et al. 2017)

### Anti-inflammatory mechanism

Natural products in psoriasis have an anti-inflammatory effect by blocking important signaling pathways like NF-κB and MAPK pathways, regulating the production of pro-inflammatory cytokines like TNF-α, IL-6, IL-17, and IL-23, and controlling the activity of immune cells like T-helper 17 (Th17) cells.

#### Curcumin

Many studies have looked at the anti-inflammatory properties of curcumin, a polyphenolic chemical extracted from the root of the turmeric plant (Curcuma longa). Through its regulation of important signaling pathways such as NF-κB and MAPK, curcumin has been demonstrated to inhibit the production of pro-inflammatory cytokines, such as TNF-α and IL-17. This regulation is useful in psoriasis for lowering inflammation and keratinocyte growth (Zhang et al. 2022). Furthermore, oral curcumin has shown to be quite successful in lowering serum IL-22 levels in psoriasis patients (Antiga et al. 2015).

#### Glycyrrhizin

Glycyrrhizin, a triterpenoid saponin extracted from the root of Glycyrrhiza glabra (licorice), has been reported to ameliorate psoriasis-like skin lesions by inhibiting TNF-α-induced inflammatory responses through the NF-κB and MAPK signaling pathways, leading to decreased production of inflammatory cytokines (Xiong et al. 2015).

#### Rosmarinic acid

Rosmarinic acid, a phenolic compound found in rosemary (Rosmarinus officinalis), has been reported to ameliorate psoriatic skin inflammation by inhibiting the production of IL-23 and IL-17A, as well as ameliorating redness and scaling (Ho et al. 2022).

#### Rhododendrin

A flavonoid glycoside called Rhododendrin, which is present in the Rhododendron genus of plants, has been demonstrated to reduce skin inflammation in mice that is similar to psoriasis and is mediated via toll-like receptor-7. Reducing cytokine production is the result of its ability to downregulate the NF-κB and MAPK signaling pathways (Jeon et al. 2017).

#### Boswellic acids

Acids derived from the resin of Boswellia serrata have demonstrated significant anti-inflammatory effects. These substances have demonstrated the ability to suppress dendritic cell activation and reduce the secretion of pro-inflammatory cytokines, including IL-12 and IL-23, which are crucial in the pathogenesis of psoriasis (Halim et al. 2020).

#### Resveratrol

Resveratrol, a polyphenolic compound found in grapes, berries, and peanuts, has been shown to exert anti-inflammatory effects by inhibiting the production of IL-17 and other inflammatory cytokines, modulating the NF-κB signaling pathway, and reducing keratinocyte proliferation in psoriasis models (Kjær et al. 2015).

### Antioxidant mechanism

Natural products exert anti-oxidant effect in psoriasis by enhancing the enzymatic activity of key antioxidants, including superoxide dismutase (SOD), glutathione (GSH), and catalase (CAT). Furthermore, these natural compounds contribute to the prevention of lipid peroxidation, primarily by lowering the levels of malondialdehyde (MDA). Additionally, they exhibit the capability to directly scavenge reactive oxygen species (ROS).

#### Green tea

There are several polyphenols in green tea (Camellia sinensis), but some of the most powerful antioxidants are catechins like epigallocatechin gallate (EGCG). Research conducted recently has shown that EGCG effectively neutralizes free radicals (Zhang et al. 2024). Also, it reduces levels of malondialdehyde (MDA) and increases activity of superoxide dismutase (SOD) and catalase (CAT), which in turn reduces inflammation similar to psoriasis caused by imiquimod in mouse models (Zhang et al. 2016).

#### Quercetin

Quercetin**,** a flavonoid found in many vegetables and fruits like apples and onions, has been shown to exert a powerful anti-oxidant effect in imiquimod-induced psoriasis, by increasing the activity of SOD, CAT, and GSH, as well as, decreasing MDA accumulation (Chen et al. 2017).

#### Carotenoids

Carotenoids, derived from cyanobacteria have demonstrated significant antioxidative properties, primarily through their ability to scavenge ROS. These compounds not only exhibit anti-oxidant effects but also possess anti-inflammatory properties, making them promising candidates for topical treatments in psoriasis (Lopes et al. 2020).

#### Salidroside

Salidroside, a glucoside derived from Rhodiola rosea, has been demonstrated to inhibit oxidative stress signaling pathways through the activation of sirtuin 1 (SIRT1), a protein that plays a critical role in regulating cellular responses to stress. By modulating key signaling pathways, including NF-κB and MAPK, salidroside effectively reduces both inflammation and oxidative damage associated with psoriatic lesions (Xu et al. 2019).

### Anti-proliferative mechanism

The anti-proliferative mechanisms of natural products in the treatment of psoriasis are primarily centered on the modulation of critical signaling pathways, including NF-κB, PI3K/Akt/mTOR, and various cell cycle regulators. Additionally, these natural compounds can induce apoptosis in hyperproliferative keratinocytes. By specifically targeting these pathways, natural products demonstrate a capacity to effectively diminish keratinocyte hyperproliferation.

#### Indigo naturalis

The active compound indirubin from Indigo naturalis has shown significant anti-proliferative effects in psoriasis models. Its mechanism of action involves the inhibition of cyclin-dependent kinases (CDKs), which results in cell cycle arrest in keratinocytes. In addition to its anti-proliferative effects, indirubin also possesses anti-inflammatory properties, thereby enhancing its therapeutic potential in the management of psoriasis (Gamret et al. 2018; He et al. 2022).

#### Delphinidin

Delphinidin is an anthocyanidin found in various pigmented fruits and vegetables. Delphinidin has been demonstrated to inhibit the PI3K/Akt/mTOR signaling pathway in imiquimod-induced psoriasis, which is frequently upregulated in psoriatic lesions and contributes to the excessive proliferation of keratinocytes (Chamcheu et al. 2017).

#### Mahonia aquifolium

The extract of *Mahonia aquifolium* (Oregon grape) has been shown to inhibit keratinocyte proliferation and reduce the production of pro-inflammatory cytokines such as IL-8. The anti-proliferative action of *Mahonia aquifolium* is attributed to its ability to modulate the NF-κB signaling pathway, thereby reducing inflammation and keratinocyte hyperproliferation (Zahir et al. 2013; Sarkar et al. 2023).

#### Artemisia capillaris

The ethanol extract of *Artemisia capillaris* has been reported to induce apoptosis in hyperproliferative keratinocytes, alleviating psoriatic symptoms (Lee et al. 2018). This mechanism is crucial in counteracting the excessive proliferation characteristic of psoriasis.

### Anti-angiogenic mechanism

Natural products exert their anti-angiogenic effect through the inhibition of key molecular targets such as vascular endothelial growth factor (VEGF) and hypoxia-inducible factor 1-alpha (HIF-1α), as well as direct effect on endothelial cells, such as human umbilical vein endothelial cells (HUVECs), inhibiting their proliferation and ability to form capillary-like structures.

#### *Qingre Huoxue* decoction

*Qingre Huoxue* decoction is a traditional herbal formulation recognized for its significant anti-angiogenic effects in psoriasis-like skin conditions. This formulation has been shown to reduce levels of key angiogenic factors, including VEGF, HIF-1α, and Flt-1. By modulating these factors, Huoxue decoction effectively contributes to decreased vascular permeability and inflammation (Li et al. 2021).

#### PSORI-CM02

PSORI-CM02 formulation consists of five herbal components: Rhizoma Curcumae, Radix *Paeoniae rubra*, *Sarcandra glabra*, *Rhizoma Smilacis glabrae*, and *Fructus mume*. It has been shown to possess anti-angiogenic efficacy both in vitro and in vivo by downregulating VEGF and HIF-1α, while also inhibiting the proliferation and tube formation of HUVECs. Through these mechanisms, PSORI-CM02 effectively disrupts the oxidative stress–inflammation–angiogenesis axis, contributing to its therapeutic potential in managing psoriasis (Lu et al. 2021).

#### Rottlerin

Rottlerin, a polyphenol natural product isolated from the Asian tree Mallotus philippensis, has been demonstrated to reduce the ability of HUVECs to form capillary-like structures, indicating its potential as an anti-angiogenic agent in psoriasis (Min et al. 2017).

## Current treatments

### Conventional therapies

#### Vitamin D

Vitamin D and its analogs are commonly employed in the management of chronic plaque psoriasis, particularly in mild-to-moderate cases. Their introduction into clinical use was based on early observations that both oral and topical forms exhibit beneficial effects on psoriatic plaques (Bhat et al. 2022). Mechanistically, vitamin D analogs, such as calcipotriol, calcitriol, and tacalcitol, act by binding to intracellular vitamin D receptors (VDR) in keratinocytes. Once activated, these receptor–ligand complexes regulate gene transcription through vitamin D response elements (VDREs), affecting pathways involved in keratinocyte proliferation, differentiation, and inflammation (Pike et al. 2012; Bikle 2014).

The genetic regulation of the VDR is implicated in psoriasis. Polymorphisms in the VDR gene are linked to altered VDR mRNA expression, which may compromise the cutaneous barrier and exacerbate psoriasis (Richetta et al. 2014). Vitamin D also modulates immune responses by downregulating inflammatory cytokines (e.g., IL-6, IL-8, TNF-α, IFN-γ) while promoting anti-inflammatory mediators such as IL-10 (Karthaus et al. 2014; Prtina et al. 2021). This immunomodulatory activity is complemented by the suppression of cyclin D and c-Myc, enhancement of intracellular calcium signaling, and inhibition of keratinocyte hyperproliferation (Svendsen et al. 1997; Bikle 2011).

To address the limitations of conventional vitamin D formulations, advanced drug delivery systems, such as nanostructured lipid carriers (NLCs) and solid lipid nanoparticles (SLNs), have been developed. These systems aim to improve skin penetration, reduce local irritation, and provide sustained drug release, thereby enhancing therapeutic outcomes and patient compliance (Lin et al. 2010).

For instance, calcipotriol-loaded NLCs, especially when co-loaded with methotrexate (CAL-MTX-NLCs), demonstrated a significant improvement in skin targeting, with enhanced delivery of methotrexate (2.4–4.4 times) and reduced calcipotriol release, mitigating the risk of irritation (Lin et al. 2010).

Similarly, dual-loaded SLNs incorporating cyclosporin and calcipotriol achieved ultra-nano sizes (< 100 nm) and high encapsulation efficiency. In vivo studies on psoriatic mouse models revealed that these NLC-based formulations outperformed both SLNs and traditional preparations in reducing skin inflammation and lesion severity (Arora et al. 2017).

Despite these promising results, nanocarrier-based formulations are still largely in the preclinical or early clinical stages. Their long-term safety, manufacturing scalability, and cost-effectiveness remain significant barriers to widespread clinical adoption. Additionally, the complexity of these systems may increase the risk of formulation instability and regulatory challenges.

#### Topical corticosteroids

Topical corticosteroids (TCS) have long been a mainstay in the treatment of mild-to-moderate psoriasis, owing to their potent anti-inflammatory, immunosuppressive, anti-proliferative, and vasoconstrictive effects. These therapeutic actions are mediated through both genomic and non-genomic pathways. Upon binding to cytoplasmic glucocorticoid receptors (GR), corticosteroids induce translocation of GR complexes into the nucleus, where they interact with glucocorticoid response elements (GREs) to upregulate anti-inflammatory genes (e.g., IL-10, DUSP-1) and suppress pro-inflammatory mediators like IL-1, IL-6, TNF-α, and chemokines, such as MCP-1 and IL-8 (McManus 2003; Zhang et al. 2009; Lee et al. 2021; Lee and Tran 2023). Simultaneously, CS suppresses the transcription of pro-inflammatory genes by inhibiting transcription factors like nuclear factor-κB (NF-κB) and activator protein 1 (AP-1), which are responsible for the expression of cytokines, chemokines, and other inflammatory mediators (Patil et al. 2018; Cacheiro-Llaguno et al. 2022; Sardana and Sachdeva 2022). Moreover, CS induces MAPK phosphatase 1 (MKP-1), which deactivates MAPKs (e.g., c-Jun), leading to reduced pro-inflammatory signaling. Defects in MKP-1 expression are associated with glucocorticoid resistance (Uva et al. 2012; Sardana and Sachdeva 2022; Milara et al. 2023).

Additionally, corticosteroids promote the expression of annexin A1, a mediator that inhibits phospholipase A2 (PLA2), thus limiting the synthesis of prostaglandins and leukotrienes (Perretti and Dalli 2009; Kwatra and Mukhopadhyay 2018). These effects help resolve inflammation, reduce keratinocyte hyperproliferation, and induce apoptosis in immune cells such as eosinophils and lymphocytes. TCS also exert vasoconstrictive effects that reduce blood flow to psoriatic lesions, limiting the influx of inflammatory cells (Kwatra and Mukhopadhyay 2018).

Despite their effectiveness, the long-term use of TCS is limited by a number of well-documented drawbacks. These include cutaneous atrophy, telangiectasia, tachyphylaxis, and in some cases, systemic effects such as adrenal suppression (Niculet et al. 2020; Chandy et al. 2023).

Clinical trials have demonstrated that while short-term use is generally safe, prolonged or inappropriate application especially of high-potency corticosteroids can result in irreversible skin damage and loss of treatment responsiveness (Lebwohl et al. 2001; Castela et al. 2012b),

To mitigate these adverse effects, combination therapies are frequently employed. For instance, pairing corticosteroids with vitamin D analogs (e.g., calcipotriol) enhances efficacy while reducing the required steroid dose and minimizing atrophy (Menter and Griffiths 2007). Additionally, agents like tazarotene (a topical retinoid), salicylic acid, and calcineurin inhibitors have been combined with corticosteroids to further modulate inflammation and keratinocyte behavior (Draelos 2022).

## Natural compounds as adjuvant enhancers in treatment of psoriasis

Given the limitations associated with both vitamin D analogs and topical corticosteroids—including the risk of cutaneous atrophy, irritation**,** tachyphylaxis**,** and limited efficacy in moderate-to-severe psoriasis—there is increasing interest in the use of natural compounds as adjunctive therapies (Bruner et al. 2003; Castela et al. 2012a; Malihi et al. 2016). Several phytochemicals, such as curcumin, resveratrol, quercetin, aloe vera**,** and tea polyphenols, have demonstrated anti-oxidant**,** anti-inflammatory**,** and immunomodulatory effects, making them promising candidates for combination strategies (Szulc-Musioł and Sarecka-Hujar 2021; Reena et al. 2022; Ebrahimi et al. 2024). When incorporated into advanced delivery systems like nanostructured lipid carriers (NLCs) or solid lipid nanoparticles (SLNs)**,** these natural agents may enhance the therapeutic efficacy of vitamin D analogs or corticosteroids by improving skin penetration**,** prolonging drug release**,** and reducing local and systemic toxicity (Bodnár et al. 2024; Burlec et al. 2024)**.** Rather than serving as standalone treatments, such natural compounds are best positioned as supportive adjuvants, helping to reduce drug dosages**,** minimize side effects, and maintain long-term disease control in chronic, relapsing forms of psoriasis.

### Curcumin

Curcumin exhibits notable anti-inflammatory and anti-oxidant properties, primarily through the inhibition of NF-κB, MAPK, and JAK/STAT pathways. Preclinical studies have demonstrated that curcumin downregulates pro-inflammatory cytokines such as TNF-α, IL-6, and IL-17, while promoting regulatory T-cell induction. A meta-analysis encompassing both clinical and preclinical studies indicated that curcumin, whether as monotherapy or in combination, improved PASI scores in patients compared to controls (Zhang et al. 2022).

Furthermore, a clinical case study highlighted the synergistic potential of combining curcumin with high-dose vitamin D therapy and an anti-inflammatory diet in managing psoriasis vulgaris. The study emphasized the modulation of the VDR as a key mechanism underlying the observed therapeutic benefits (Beltran and Guimarães., 2023).

### Resveratrol

Resveratrol possesses anti-proliferative and anti-inflammatory effects by suppressing IL-17A, TNF-α, and COX-2, and activating SIRT1 signaling pathways. In vitro studies have shown that resveratrol attenuates inflammation and promotes keratinocyte differentiation (Wen et al. 2020).

Elgewelly et al. (2022) developed a resveratrol-loaded vesicular elastic nanocarrier gel and evaluated its efficacy in an imiquimod-induced psoriasis mouse model. The formulation demonstrated significant anti-psoriatic effects, including reduced epidermal hyperplasia, erythema**,** and inflammatory infiltration**,** as well as favorable in vitro permeation and skin deposition profiles. These results emphasize the role of advanced vesicular delivery systems in enhancing the therapeutic effectiveness of resveratrol for topical psoriasis treatment. (Elgewelly et al. 2022).

### Quercetin

Quercetin exhibits strong anti-oxidant and anti-inflammatory properties. It downregulates pro-inflammatory cytokines, including IL-1β, IL-6, and TNF-α, and inhibits NF-κB activity. Animal studies have demonstrated that quercetin supplementation significantly reduces levels of TNF-α, IL-6, and IL-17, thereby alleviating psoriasis symptoms (Zou et al. 2024).

Additionally, the development of quercetin-loaded phytosomal hydrogels has been explored to enhance skin delivery and stability, indicating potential for co-formulation with vitamin D analogs in psoriasis therapy (Ahmad and Tiwari 2025).

### Aloe vera

Aloe vera gel is renowned for its wound healing, anti-inflammatory, and immunomodulatory effects, attributed to bioactive compounds like acemannan and aloenin. A prospective, randomized clinical trial compared the efficacy of topical aloe vera with 0.1% triamcinolone acetonide in patients with mild-to-moderate plaque psoriasis. The study found that aloe vera was at least as effective as the corticosteroid in reducing psoriatic plaques, with a significantly greater reduction in the PASI scores (Choonhakarn et al. 2010).

### Epigallocatechin gallate (EGCG)

EGCG, the predominant polyphenol in green tea, has demonstrated efficacy in inhibiting keratinocyte proliferation and modulating Th1/Th17 immune responses. In murine models, topical application of EGCG significantly reduced psoriasiform inflammation and normalized skin architecture (Zhang et al. 2016).

Furthermore, chitosan-based nanoformulations of EGCG have been developed to enhance its stability and skin penetration, offering a promising approach for topical psoriasis therapy (Chamcheu et al. 2018).

## Comparisons between natural agents and standard pharmacological treatments

- **Turmeric (Curcuma longa)** vs **Clobetasol Propionate**: In a randomized-controlled trial, topical 1% turmeric cream showed non-inferior efficacy to 0.05% clobetasol in reducing PASI and pruritus scores over 4 weeks, with fewer adverse events reported in the turmeric group, indicating better tolerability (Guevara et al. 2019).
- **Nigella sativa (black seed)** alone and in combination with **methotrexate** was found to reduce PASI scores and relapse rates significantly. In a study by Ahmed et al. (2014), the combination of oral and topical *Nigella sativa* with methotrexate resulted in 90% marked clinical response with only 27% relapse, versus 56% relapse in the methotrexate-alone group. Notably, Nigella sativa also reduced liver enzyme elevations, suggesting a hepatoprotective effect.
- **Glycyrrhizin** combined with **acitretin** improved PASI scores and reduced liver toxicity. Yu et al. (2020) reported that in patients with generalized pustular psoriasis and liver test abnormalities, the addition of glycyrrhizin led to significant clinical improvement and normalized liver function within 2 weeks. This was sustained over a 12-month follow-up, supporting both enhanced efficacy and safety.
- **Aloe vera** vs **triamcinolone acetonide**: In a comparative trial by (Choonhakarn et al. 2010), topical Aloe vera cream was as effective as 0.1% triamcinolone in reducing PASI and DLQI scores in mild-to-moderate psoriasis but was associated with fewer adverse effects, highlighting its favorable safety profile for long-term use.

## The regulatory status and safety considerations of key natural products:

Several of the natural products discussed in our review are already included in national pharmacopoeias or approved for over-the-counter (OTC) use, particularly in the form of dietary supplements or topical agents:

- **Curcumin** from (*Curcuma longa*) and **Nigella sativa oil** are classified as generally recognized as safe (GRAS) by the United States Food and Drug Administration (FDA) for use in food products and dietary supplements (Sharifi-Rad et al. 2020; Burdock 2022)
- **Aloe vera gel**, **glycyrrhizin** from (*Glycyrrhiza glabra*), and **boswellic acids** from (*Boswellia serrata*) are permitted as active ingredients in licensed herbal medicines or cosmeceuticals by regulatory bodies such as:
  o The **European Medicines Agency (EMA)** under its traditional herbal medicinal product scheme (e.g., EMA/HMPC/5829/2010 for *Aloe vera*) (In 2017).
  o The **Chinese Pharmacopoeia**, which includes glycyrrhizin and *Boswellia* in traditional medicine formulas (State Pharmacopoeia Commission of the PRC, 2020)
- **Boswellia serrata and Glycyrrhiza glabra** were recognized by the **German Commission E monographs** for treating inflammatory conditions, including arthritis, bronchitis, and skin inflammation (Nathan and Scholten 1999).

However, it is important to note that **none of the phytochemicals reviewed are currently approved as monotherapies for psoriasis by major regulatory agencies** such as the **FDA** or **EMA**. Most are used as supportive or complementary therapies. We emphasize that further **randomized-controlled trials (RCTs)** with rigorous pharmacokinetic and safety profiling are necessary to advance regulatory approval for dermatologic indications such as psoriasis.

## Conclusions

Of all the immune-mediated diseases, psoriasis is among the most common and unfortunately incurable. Nowadays, there are a plethora of treatment choices available because of the abundance of clinical research investigations. Despite their usefulness in alleviating symptoms, many medications do not heal the underlying illness and can even cause serious adverse effects. The many impacts of natural products on the inflammation and immunological dysregulation that underline psoriasis make them a promising treatment option. They manage inflammatory cytokines, slow the development of keratinocytes, and limit the activity of Th17 cells, which in turn reduces inflammation, redness, scaling, and irritation. In addition to complementing conventional therapies, these methods promote skin healing by calming the skin and boosting anti-oxidant defenses. Their ability to augment current therapies while also reducing the risk of adverse effects highlights their promise for improving patient outcomes in psoriasis therapy. Targeted therapy and precision medicine that zero in on certain cells or genes are necessities in this age of precision medicine, although these therapies are mostly for mild-to-severe psoriasis. To effectively treat psoriasis, further research is required.

## Data Availability

No datasets were generated or analyzed during the current study.
